# Discrete Time Series Forecasting in Non-Invasive Monitoring of Managed Honey Bee Colonies: Part II: Are Hive Weight and In-Hive Temperature Seasonal and Colony-Specific?

**DOI:** 10.3390/s25144319

**Published:** 2025-07-10

**Authors:** Vladimir A. Kulyukin, Aleksey V. Kulyukin, William G. Meikle

**Affiliations:** 1 Department of Computer Science, Utah State University, Logan, UT 84322, USA; 2 Department of Mathematics and Statistics, Utah State University, Logan, UT 84322, USA; 3 Carl Hayden Bee Research Center, USDA-ARS, Tucson, AZ 85719, USA; william.meikle@usda.gov

**Keywords:** precision apiculture, continuous beehive monitoring, electronic beehive monitoring, sensor-based hive monitoring, time series forecasting, ARIMA, SARIMA, hive weight and in-hive temperature datasets

## Abstract

We explored the stationarity, trend, and seasonality of the hive weight and in-hive temperature of ten managed honey bee (*Apis mellifera*) colonies at a research apiary of the Carl Hayden Bee Research Center in Tucson, Arizona, USA. The hives were monitored with electronic scales and in-hive temperature sensors from June to October 2022. The weight and temperature were recorded every five minutes around the clock. The collected data were curated into 2160 timestamped weight and 2160 timestamped temperature observations. We performed a systematic autoregressive integrated moving average (ARIMA) time series analysis to answer three fundamental questions: (a) Does seasonality matter in the ARIMA forecasting of hive weight and in-hive temperature? (b) To what extent do the best forecasters of one hive generalize to other hives? and (c) Which time series type (i.e., hive weight or in-hive temperature) is better predictable? Our principal findings were as follows: (1) The hive weight and in-hive temperature series were not white noise, were not normally distributed, and, for most hives, were not difference- or trend-stationary; (2) Seasonality matters, in that seasonal ARIMA (SARIMA) forecasters outperformed their ARIMA counterparts on the curated dataset; (3) The best hive weight and in-hive temperature forecasters of the ten monitored colonies appeared to be colony-specific; (4) The accuracy of the hive weight forecasts was consistently higher than that of the in-hive temperature forecasts; (5) The weight and temperature forecasts exhibited common qualitative patterns.

## 1. Introduction

Managing honey bee (*Apis mellifera*) colonies requires that human bee keepers conduct regular hive inspections and treatments, and travel to distant apiaries. Sensor-based monitoring alleviates some well-known bottlenecks in human monitoring such as limited time, impatience, and vehicle wear and tear. It also provides consistent information on various aspects of the colony’s phenology and helps with the retroactive identification of performance bifurcation points. As off-the-shelf electronic sensors become more reliable and smaller in size, they will be increasingly integrated into multisensor hive monitoring systems [1,2,3,4]. Thus, stakeholders of multisensor hive monitors must choose not only which sensors to use and when, but also which software works best with their selection [5].

Two sensors that have played a critical role in continuous hive monitoring are electronic scales and in-hive temperature sensors [6,7]. Scales have become popular among precision apiculture researchers and practitioners because they generate regular weight observations around the clock, regardless of ambient weather, and do not interfere with hive inspections. Foraging activity is reflected by weight changes due to forager traffic, and foraging success can be estimated with increases in hive food stores [8]. However, scales require initial calibration and subsequent recalibration and are hard to move from hive to hive and apiary to apiary. Unlike scales, in-hive temperature sensors are smaller in size, less expensive, and have smaller power footprints, which makes them more mobile and deployable than scales. In-hive temperature observations are indicative of a colony’s thermoregulatory behaviors, e.g., clustering and fanning, and can be correlated with the diurnal rhythms of metabolic rate and activity [9]. However, due to their small size, in-hive temperature sensors are easy to lose or damage during hive inspections or hive woodenware maintenance.

Hive weight has been used in precision apiculture studies to characterize different events in a colony’s biological life cycle (e.g., honey production [10], swarming and colony abandonment [11], overwintering [12], pesticide exposure [13], and proximity to conservation reserve lands [14]). In-hive temperature has also been used to investigate colony phenology (e.g., daily metabolic cycles [15], colony size [16], and thermoregulation as a function of subspecies [17] or genetic diversity [18]). These studies provided valuable insights into the relationship of a colony’s phenology to hive weight and in-hive temperature. However, due to the fundamentally descriptive nature of the employed techniques of regression and detrending, they formulated and tested specific hypotheses or classified static events, but did not attempt to forecast into the future. This gap, which has been recently noted in the precision apiculture literature [1,5], can be formulated as follows: computational models that forecast the status of managed honey bee colonies using affordable off-the-shelf sensors are few and far between.

A key objective of our investigation was to address this gap by investigating time series forecasters of hive weight and in-hive temperature observations that researchers and practitioners can obtain with off-the-shelf sensors. Specifically, we explored and exploited the forecasting of hive weight and in-hive temperature with ARIMA and SARIMA over longer forecast horizons (several hundred hours). We are not aware of such investigations in the precision apiculture literature. Our contributions to precision apiculture and sensor-based hive monitoring reported in this article are as follows:

(1) An exploration of the stationarity, trend, and seasonality of hive weight and in-hive temperature time series with the Ljung–Box White Noise test [19], the Augmented Dickey–Fuller test [20], the Kwiatkowski–Phillips–Schmidt–Shin test [21], and the Shapiro–Wilk Normality test [22];
(2) A systematic exploitation of a curated dataset with a grid search of 1538 (769 weight and 769 temperature) ARIMA/SARIMA forecasters to answer three fundamental questions: (a) Does seasonality matter in the ARIMA forecasting of hive weight and in-hive temperature? (b) To what extent do the best forecasters of one hive generalize to other hives? and (c) Which time series type (i.e., hive weight or in-hive temperature) is better predictable?
(3) Three principal findings were as follows: (a) seasonality matters, in that SARIMA forecasters outperformed their ARIMA counterparts; (b) the best hive weight and in-hive temperature forecasters of the ten monitored colonies appeared to be colony-specific; and (c) hive weight was more predictable than in-hive temperature.
(4) Our grid search ARIMA/SARIMA source code and the top hive weight and in-hive temperature forecasters found using it can be used by researchers, practitioners, and citizen scientists in the longitudinal monitoring of managed honey bee colonies across diverse environments [23].
(5) A curated dataset of 4320 timestamped observations (2160 hive weight and 2160 in-hive temperature) from ten managed honey bee colonies at a United States Department of Agriculture (USDA) research apiary [23].

The remainder of our article is organized as follows: In Section 2, we review related research. In Section 3, we describe our metadata, data, and methods. In Section 4, we report the results of our investigation. In Section 5, we discuss our results, and the implications and limitations of our findings. In Section 6, we offer our conclusions and outline the scope of our future work.

## 2. Related Work

Hive weight and in-hive temperature observations have been used in many studies of colony phenology. Thoenes and Buchmann [11] showed that colony weight is related to foraging, swarming, and hive abandonment. Marceau et al. [10] demonstrated a regressive, polynomial relationship between hive weight and colony growth and productivity. Meikle et al. [24] investigated how colony weight may be related to pesticide exposure. Stalidzans et al. [12] reported a relationship between colony weight and overwintering. Szabo et al. [15] reported that when in-hive temperature sensors were placed inside or close to the cluster of bees, i.e., the mass of bees at the core of the colony, the temperature readings were more affected by the cluster than by exterior conditions. The experiments by Southwick and Moritz [25] showed that daily cycles of in-hive temperature and metabolic activity may be driven by ambient weather. Meikle et al. [16] reported some evidence of the thermoregulation of colonies in the absence of brood, and that in-hive temperature may be affected by colony size and the location of the in-hive temperature sensor. Worswick [17] argued that a colony’s thermoregulation was a function of subspecies. Jones et al. [18] found evidence that thermoregulation may be related to within-colony genetic diversity. Stalidzans and Berzonis [26] offered some evidence that thermoregulation may be a function of a colony’s biological life cycles.

A notable recent trend in precision apiculture is predictive sensor-based monitoring of managed colonies. In a 3-year long investigation of multiple colonies at different geographical locations, Braga et al. [1] used in-hive temperature, hive weight, ambient temperature, dew point, wind direction, wind speed, rainfall, and daylight in combination with weekly apiary inspection results. The team designed K-nearest neighbors (KNNs) models, random forests, and artificial neural networks to assess colony health from in-hive temperature, hive weight, and ambient weather. On a curated dataset, random forest turned out to be the best predictor of hive health, with an accuracy of 98%. Zaman and Dorin (2023) [5] proposed a comprehensive qualitative framework to assess predictive hive monitoring systems from the viewpoints of different stakeholders, e.g., research institutions, regulatory agencies, and commercial operations. Kulyukin et al. [27] compared non-seasonal ARIMA with artificial, convolutional, and long short-term memory networks as time series forecasters of hive weight, in-hive temperature, and omnidirectional forager traffic at the hive’s entrance over the short forecast horizons of 6, 12, 18, and 24 h. Non-seasonal ARIMA performed on par with machine learning counterparts in predicting falling, rising, and unchanging trends.

There is evidence of predictive sensor-based modeling on the Internet sites of several commercial platforms. Thus, *Arnia* (https://arnia.co/ accessed on 5 May 2025) claims to provide predictive modeling on the basis of eight sensors: audio, temperature, humidity, weight, light sensor, accelerometer, bee counter, and video; *ApisProtect* (https://pitchbook.com/profiles/company/182117-80 accessed on 5 May 2025) states that its hive monitoring tools use four sensors: temperature, humidity, audio, and accelerometer; *IOBee* (https://io-bee.eu/ accessed on 5 May 2025) also uses temperature, humidity, weight, and bee counts to assess colony status; *Pollenity* (https://www.pollenity.com/ accessed on 5 May 2025) proposes a hive monitoring system using temperature, humidity, weight, and acoustic sensors. The actual algorithms and datasets of these commercial platforms appear to be proprietary, which is understandable, because their business models likely depend on them.

## 3. Materials and Methods

### 3.1. Metadata and Data

**Figure A1:**
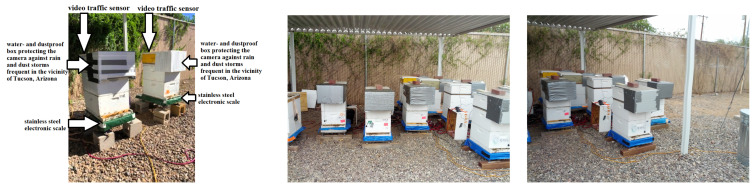
**Sensor placement and research apiary.** Sensor placement in a monitored hive (**left**): weight and video sensors (not used in this study) are external; temperature sensor—internal. Two views ((**middle**) and (**right**)) of the USDA-ARS research apiary in Tucson, AZ, USA (GPS coordinates: 32°13′18.274″ N, 110°55′35.324″ W) where the hive weight, in-hive temperature, and video entrance traffic data were collected from 10 hives from June to October, 2022. The large light gray boxes on the monitored hives protected cameras against the elements. The PDF should be enlarged to see the details.

The data were acquired from ten managed colonies at a research apiary of the Carl Hayden Bee Research Center of the U.S. Department of Agriculture Agricultural Research Service (USDA-ARS) in Tucson, AZ, USA (GPS coordinates: 32°13′18.274″ N, 110°55′35.324″ W) from June to October 2022. Each hive included a bottom board with a landing pad, two deep Langstroth boxes with ten frames in each, an inner hive cover, a box with an on-hive camera, and a hive cover with a cardboard box reinforced with all-weather duct tape to protect the camera against the elements. Each hive was mounted on an electronic scale and an in-hive temperature sensor was placed at the top bar of the middle frame in the second deep super of each hive (cf. Figure A1).

For each hive, 2160 hive weight and 2160 in-hive temperature observations were timestamped and logged. No observations were missed due to sensor failures. The weight and temperature observations were smoothed by computing hourly means. All hourly means were labeled with natural numbers to obtain a universal time axis for the time series analysis. The curated dataset is provided in the supplementary materials [23]. Hourly means were used as the ground truth observations of the hive weight time series, denoted as $\left\{W_{t}\right\}$, and of the in-hive temperature time series, denoted as $\left\{C_{t}\right\}$. The notation $\left\{C_{t}\right\}$ was chosen because the temperature observations were logged in degrees Celsius (Table 1).

Five hives (USDA-ARS IDs 2059, 2120, 2141, 2142, 2158) had Russian queens and five hives (USDA-ARS IDs 2123, 2129, 2130, 2137, 2146)—Wooten. Hive evaluations were conducted on 21 June, 11 August, and 23 September. Each evaluation included a visual queen status check (presence/absence) and removal of queen supersedure cells. On 20 June 2022, 2141 was requeened; 2140 was requeened on 23 June 2022. On 22 July 2022, a supersedure queen cell was removed from 2059. On 26 July 2022, 2141 and 2059 were requeened. On 15 August 2022, a laying worker was detected in 2141. On 19 September 2022, the queens were removed from 2158 and 2120 for unrelated, scheduled experiments. The final hive evaluations were conducted on 23 September 2022.

Adult bee mass in kg was determined by subtracting the weight of the frames and woodenware from the total hive weight as measured the previous midnight. Digital frame photographs were taken during hive evaluations and analyzed for brood surface area. The areas of sealed brood per frame were summed across all frames from that hive to provide a brood surface area estimate for the colony. Thirty-second videos of forager traffic at the hive’s entrance were recorded every five minutes from 7:00 to 20:55 daily. The analysis of adult bee mass, frame photographs, and forager traffic videos are outside of the scope of this investigation.

### 3.2. Time Series Forecasting

Auto-Regressive Integrated Moving Average (ARIMA) models were originally developed by Wiener in the 1940s for electrical engineering applications [28] and were applied by Box, Jenkins, and Riensel to business and economic data in the 1970s [29]. ARIMA models a time series through self (auto)-regression (i.e., looking at the previous values in the same time series). The prediction ${\hat{Y}}_{t}$ of a random variable $Y_{t}$ is the sum of an optional constant, a weighted sum of a finite number of recent values (the AR component), and a weighted sum of a finite number of recent errors (the MA component). Thus, ${\hat{Y}}_{t}=C+AR\left(p\right)+MA\left(q\right)$, where *C* is an optional real constant, *p* is the number (a non-negative integer) of terms in the weighted sum of the recent values of the series, and *q* is the number (a non-negative integer) of terms in the weighted sum of the recent errors. The parameters *p* and *q* designate the numbers of autoregressive and moving average terms, respectively.

A time series is *stationary* if its standard statistical properties (e.g., mean, standard deviation, variance, etc.) stay more or less constant over time. In practice, stationary time series tend to be more predictable, because they exhibit little trend or heteroskedasticity (i.e., heterogeneity of variance). Differencing is a technique used to stationarize a series. A differenced series is called *integrated* (hence, incidentally, the I in ARIMA). The parameter *d* specifies the number of non-seasonal differences. If we denote the original time series as $Y_{t}$ and the differenced (i.e., stationarized) series as $y_{t}$, then, if d=0 (i.e., no differencing), the prediction is ${\hat{Y}}_{t}=y_{t}=Y_{t}$, i.e., the prediction is the value of the current measurement. If d=1, the prediction is ${\hat{Y}}_{t}=y_{t}=Y_{t}-Y_{t-1}$. If d=2, the prediction is ${\hat{Y}}_{t}=y_{t}$ = $\left(Y_{t}-Y_{t-1}\right)$− $\left(Y_{t-1}-Y_{t-2}\right)$ = $Y_{t}-2Y_{t-1}+Y_{t+2}$≠$Y_{t}-Y_{t-2}$. Thus, the second difference is a measure of acceleration. The integers 1, 2, *…* in $Y_{t-1}$, $Y_{t-2}$, *…* are called *lags* and denote how many steps back are taken along a time axis. A complete ARIMA model is denoted as ARIMA(*p*, *d*, *q*), where *p* is the number of AR lags (i.e., AR(p)), *q* is the number of MA lags (i.e., MA(q)), and *d* is the differencing factor. The construction of an ARIMA model begins by stationarizing the series, if necessary, through differencing and possibly averaging, logging, deflating, or other smoothing data transformations. Autocorrelations and partial autocorrelations are examined to determine whether lags in the stationarized series or lags in the forecast errors should be incorporated into the forecast. The model suggested by this analysis is fitted, and its residual diagnostics, i.e., the residual autocorrelation function (ACF) and partial autocorrelation function (PACF) plots are examined to ensure that all coefficients are significant.

In the early 2000’s, seasonal ARIMA (SARIMA) was created, to include seasonality in time series forecasting [30]. The seasonal component is characterized by three additional parameters: *P* (the number of seasonal autoregressive terms); *D* (the number of seasonal differences); and *Q* (the number of seasonal moving-average terms). A complete SARIMA model is given as SARIMA (p,d,q,P,D,Q,S), where *p*, *d*, *q* are non-seasonal ARIMA parameters; P,D,Q are the seasonal equivalents of *p*, *d*, and *q*, respectively; and *S* is the seasonal period (e.g., S=24 for hourly data). Time series stationarity is achieved by combining the non-seasonal and seasonal differences. For example, if d=0 and D=1, then ${\hat{Y}}_{t}=y_{t}=Y_{t}-Y_{t-S}$, whereas if d=1 and D=1, then ${\hat{Y}}_{t}=y_{t}$ = $\left(Y_{t}-Y_{t-1}\right)-\left(Y_{t-S}-Y_{t-S-1}\right)$. The introduction of the seasonal terms SAR(*P*) and SMA(*Q*) obviously adds complexity, insomuch as when fitting a model to a series with a pronounced seasonal pattern, various combinations of non-seasonal and seasonal differences must be tried and the ACF and PACF plots examined at multiples of *S* to determine the number of the SAR and SMA terms in the forecasting equation.

### 3.3. Exploration

Four time series tests were applied to explore the hive weight and in-hive temperature series and to inform the subsequent grid search of optimal ARIMA and SARIMA models: (1) Ljung–Box White Noise [19]; (2) Augmented Dickey–Fuller [20]; (3) Kwiatkowski–Phillips–Schmidt–Shin [21]; and (4) Shapiro–Wilk Normality [22]. All tests were executed at α=0.05 with PyCaret (https://pycaret.org accessed on 17 June 2025), a low- code machine learning Python library.

The hypotheses of Ljung–Box White Noise are as follows: the null hypothesis (H0) is that the time series data are white noise (i.e., the data are random and there is no autocorrelation); the alternative hypothesis (H1) is that the time series data are not white noise. The hypotheses of Augmented Dickey–Fuller (ADF) are as follows: H0 is that the time series data are not difference-stationary; H1 is that the time series data are difference-stationary. The hypotheses of Kwiatkowski–Phillips–Schmidt–Shin (KPSS) are as follows: H0 is that the time series data are trend-stationary; H1 is that the time series data are not trend-stationary. The hypotheses of Shapiro–Wilk Normality are as follows: H0 is that the time series data are normally distributed; H1 is that the time series data are not normally distributed.

### 3.4. Forecaster Grid Search and Generalizability

**Figure 1:**
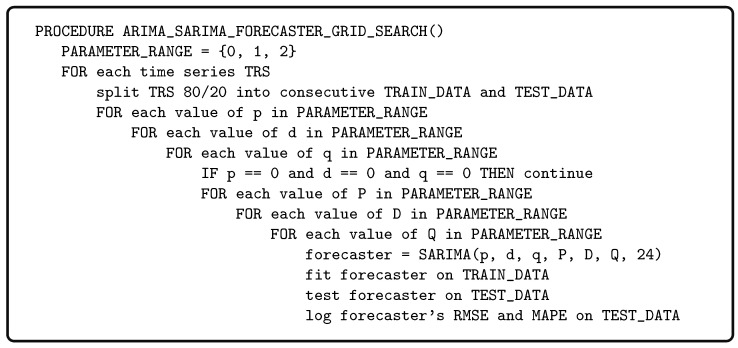
**ARIMA and SARIMA forecaster grid search.** The pseudocode of the forecaster grid search procedure. The actual Python code is given in the supplementary materials [23].

The parameter ranges in the grid search were informed by the findings in [31]. We implemented the grid search in Python 3.10.12 using PyCaret 3.3.2 and executed it on a Hewlett Packard Z220 workstation with Ubuntu 22.10. Figure 1 gives the pseudocode of the grid search procedure. The grid search examined all SARIMA (p,d,q,P,D,Q,S) forecasters, where 0≤p,d,q,P,D,Q≤2 and S=24, i.e., the seasonality was fixed at 24 h. The CSV processing was performed with pandas 2.1.4 (https://pandas.pydata.org accessed on 17 June 2025). The plots were generated with matplotlib 3.7.5 (https://matplotlib.org/ accessed on 17 June 2025). RMSE and MAPE were computed with scikit-learn 1.4.2 (https://scikit-learn.org accessed on 17 June 2025) and numpy 1.26.4 (https://numpy.org accessed on 17 June 2025).

A total of 729 weight and 729 temperature forecasters were fitted and evaluated with RMSE and MAPE in the grid search. The number 729 comes from the parameter ranges 0≤p,d,q,P,D,Q≤2 used in the grid search. Each of the six parameters can take on the values of 0, 1, and 2. Hence, there were $3^{6}=729$ possible forecasters. The supplementary materials [23] include plots of the second- and third-best native hive weight and in-hive temperature forecasters for each hive.

There were ten hive weight and ten in-hive temperature time series, one for each hive. In our implementation, we designated these as MWHID or MTHID, where MWH and MTH abbreviate *mean weight of hive* and *mean temperature of hive*, respectively, and ID designates the USDA-ARS hive id in Tucson, AZ. For example, MWH2059 and MTH2059 are the hive weight and in-hive temperature time series for hive 2059. Each time series had 2160 observations and was split 80/20 into training (1728 consecutive observations) and testing (the remaining 432 consecutive observations) datasets. All forecasters were fitted on the training dataset and tested on the testing dataset. The performance of each fitted forecaster on the testing dataset was evaluated with the root mean squared error (RMSE) and with the mean absolute percentage error (MAPE).

In analyzing the performance of the top forecasters, we distinguished *native* and *non-native* forecasts. A forecast for hive *H* was native to a forecaster *F* if, and only if, *F* was fitted on the training data from *H* and evaluated with RMSE or MAPE on the test data from *H*. We also refer to *H* as the *native hive* of *F*. Thus, the native forecasts indicated how well a forecaster predicted a characteristic (weight or temperature) of a hive on whose training data it was fitted. We ranked all grid-searched forecasters by RMSE and MAPE on the test data of their native hives, from smallest to largest, to identify the top three forecasters for each hive. Our objective here was to compare RMSE and MAPE as forecaster selection metrics. We will hereafter refer to a top forecaster found with RMSE as an *RMSE forecaster* and with MAPE as a *MAPE forecaster*. We applied the top native forecaster of each hive to the test data from the nine other hives, in order to estimate the generalizability of the ARIMA/SARIMA forecasters. We will call plots of non-native forecasts *generalizability plots*, because they reflect, to a degree, how well forecasters generalized to non-native hives. As we analyzed the forecast plots, we attempted to discover weight and temperature forecast patterns to analyze forecasts qualitatively. After patterns were identified, we counted their distributions in the native and non-native forecasts.

## 4. Results

### 4.1. Exploration

Table 2 contains a summary of the hive weight exploration. The seasonality was multiplicative for all hives. The primary seasonality was 24 for all hives, except for 2141, for which it was estimated at 13. The recommended differencing *d* was 1 for all hives, except for 2142, for which it was estimated at 2. The recommended seasonal differencing *D* was 0 for all hives. Ljung–Box White Noise was false for all hives, which indicated that no weight time series were white noise and justified our subsequent grid search of hive weight forecasters. ADF was false for all hives, except for 2142, indicating that 2142 was estimated to be the only hive whose weight time series was difference-stationary. KPSS was false for all hives, indicating that no weight time series were trend-stationary. Shapiro–Wilk Normality was false for all hives, indicating that no weight time series were normally distributed.

Table 3 contains a summary of the in-hive temperature exploration. The seasonality was multiplicative for all hives. The primary seasonality was 24 for all hives, except for 2141, for which it was estimated to be 48. The recommended differencing *d* was 1 for all hives. The recommended seasonal differencing *D* was 0 for all hives. Ljung–Box White Noise was false for all hives, indicating that no temperature time series were white noise. Thus, our subsequent grid search of temperature forecasters was justified. ADF indicated that the series of 2120, 2123, 2137, 2142, and 2146 were not difference-stationary, whereas 2059, 2129, 2130, 2141, and 2158 were difference-stationary. KPSS indicated that no temperature series were trend-stationary, except for 2130. Shapiro–Wilk Normality was false for all hives, indicating that no temperature time series were normally distributed.

### 4.2. Forecaster Grid Search and Generalizability

**Figure A3:**
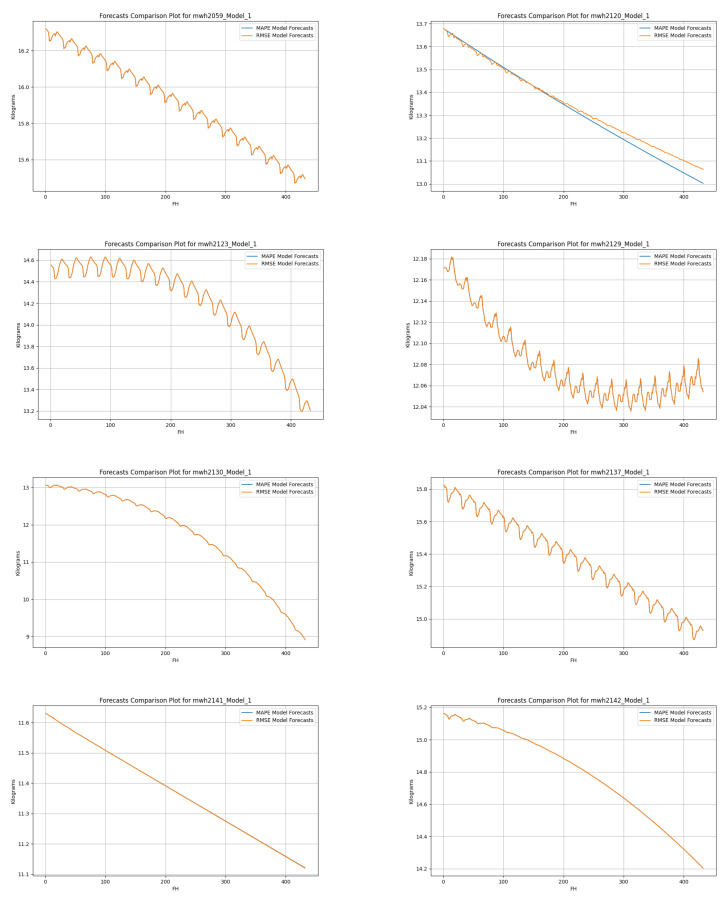

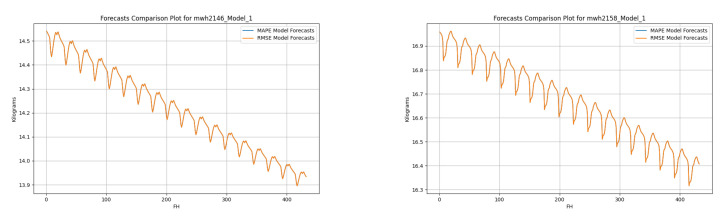
**RMSE vs. MAPE of the top hive weight forecasters of each hive.** Each of the ten plots (one per hive) has two curves: the first curve is the prediction curve of the best RMSE weight forecaster for the hive, i.e., 432 mean hour weight observations; the second curve is the prediction curve of the best MAPE weight forecaster for the same hive. The PDF should be enlarged to see the details.

**Figure A4:**
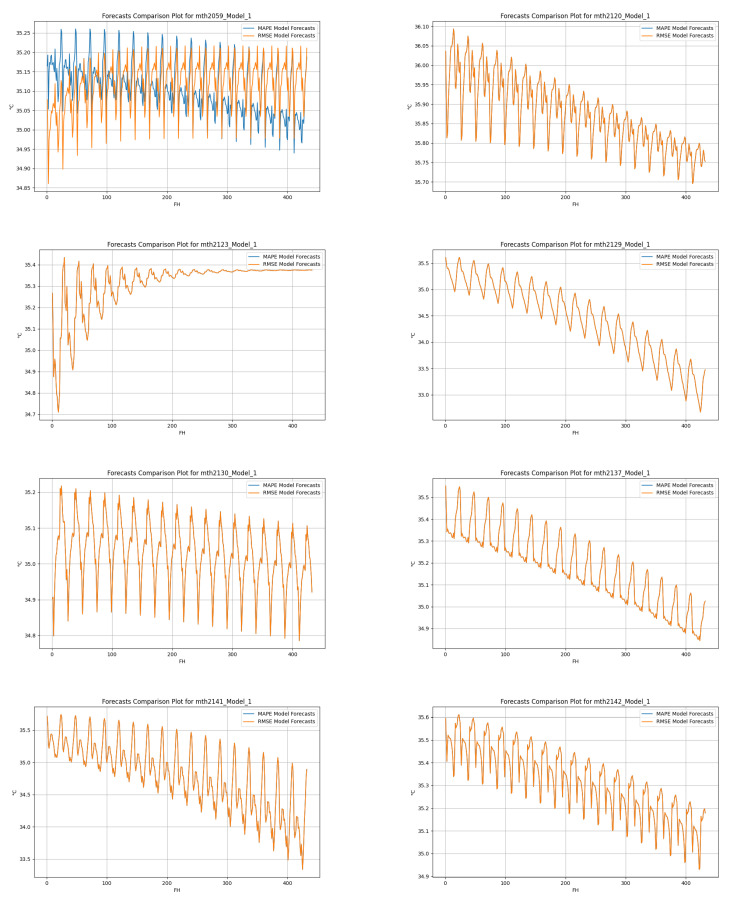

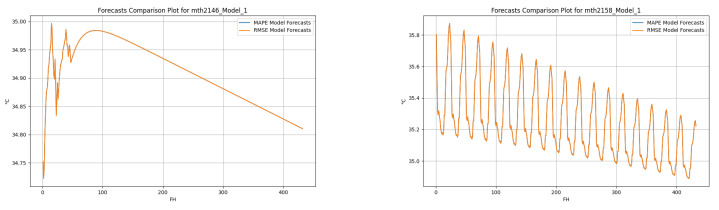
**RMSE vs. MAPE of the top in-hive temperature forecasters of each hive.** Each of the ten plots (one per hive) has two curves: one curve is the prediction curve of the best RMSE in-hive temperature forecaster for the hive, i.e., 432 mean hour in-hive temperature observations; the second curve is the prediction curve of the best MAPE in-hive temperature forecaster for the same hive. The PDF should be enlarged to see the details.

Table 4 lists the top three native RMSE and MAPE forecasters for each hive found in the grid search. Figure A3 and Figure A4 give plots (one plot per hive) that compare the complete prediction curves (i.e., 432 observations) of the best (top 1) hive weight and in-hive temperature forecasters, one chosen by RMSE and the other by MAPE. The supplementary materials [23] include plots of the second- and third-best native hive weight and in-hive temperature forecasters for each hive.

#### 4.2.1. Weight Forecasts

Table 4 gives the top three native RMSE and MAPE weight forecasters for each hive found in the grid search. Figure A16 gives generalizability plots of the top RMSE 2059 hive weight forecaster on the test data of all ten hives. Figure A17 gives generalizability plots of the top MAPE 2059 weight forecaster on test data of all ten hives. The supplementary materials give analogous generalizability plots of the other nine top native weight forecasters.

**Figure A5:**
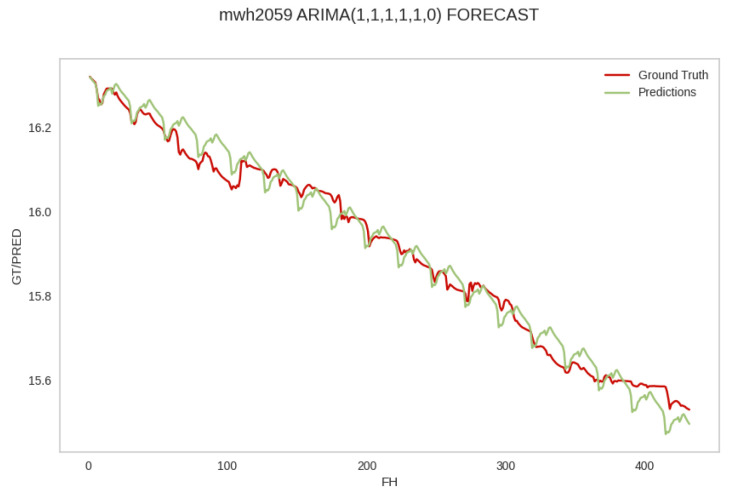
**Pattern 1: Coincidence.** The performance of the top RMSE forecaster p=1, d=1, q=1, P=1, D=1, Q=0 for hive 2059. The forecast values coincide, more or less closely, with the ground truth observations and reflect the ground truth trend. The abbreviation *mwh* in the plot stands for *mean weight of hive*. The PDF may have to be enlarged to see the plot legends.

**Figure A6:**
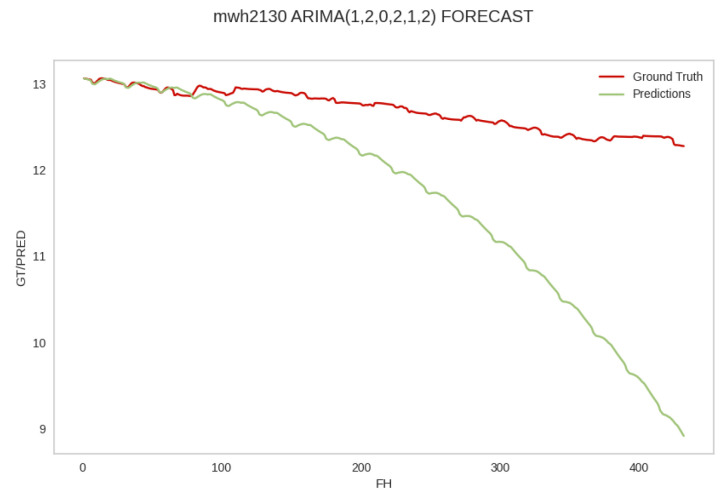
**Pattern 2: partial coincidence.** The performance of the top MAPE forecaster p=1, d=2, q=0, P=2, D=1, Q=2 for hive 2130 on the test data for the same hive. The forecast values coincide, more or less closely, with the ground truth values and reflect the ground truth trend for an initial segment of the forecast horizon but then deviate from the trend and underestimate the ground truth observations. The abbreviation *mwh* in the plot stands for *mean weight of hive*. The PDF may have to be enlarged to see the plot legends.

**Figure A7:**
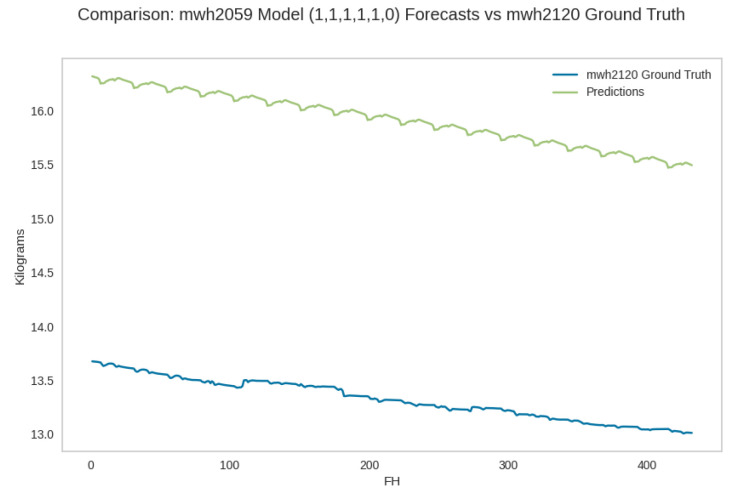
**Pattern 3: trend coincidence.** The performance of the top RMSE forecaster p=1, d=1, q=1, P=1, D=1, Q=0 for hive 2059 on the test data from hive 2120. The forecast values reflect the ground truth trend, but overestimate the ground truth observations. The abbreviation *mwh* in the plot stands for *mean weight of hive*. The PDF may have to be enlarged to see the plot legends.

**Figure A8:**
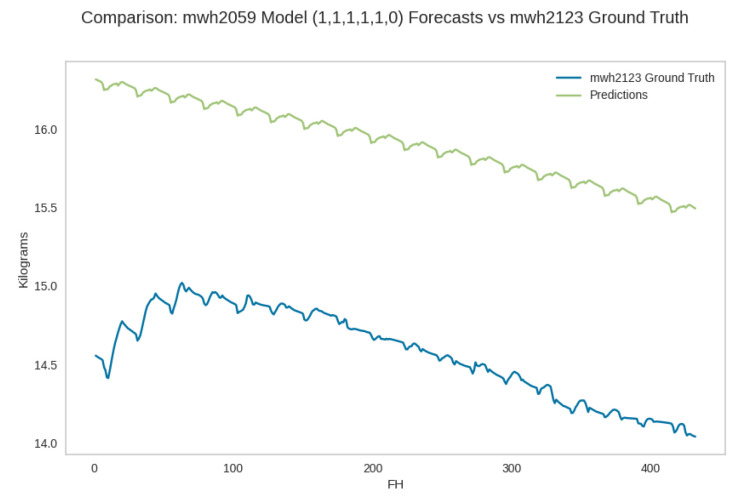
**Pattern 4: partial trend failure and coincidence.** The performance of the top RMSE forecaster p=1, d=1, q=1, P=1, D=1, Q=0 for hive 2059 on the test data for hive 2120. The forecast values fail to reflect the trend for the initial segment of the forecast horizon but reflect the trend for the remainder of the horizon; however, the forecast values overestimate the ground truth observations. The abbreviation *mwh* in the plot stands for *mean weight of hive*. The PDF may have to be enlarged to see the plot legends.

**Figure A9:**
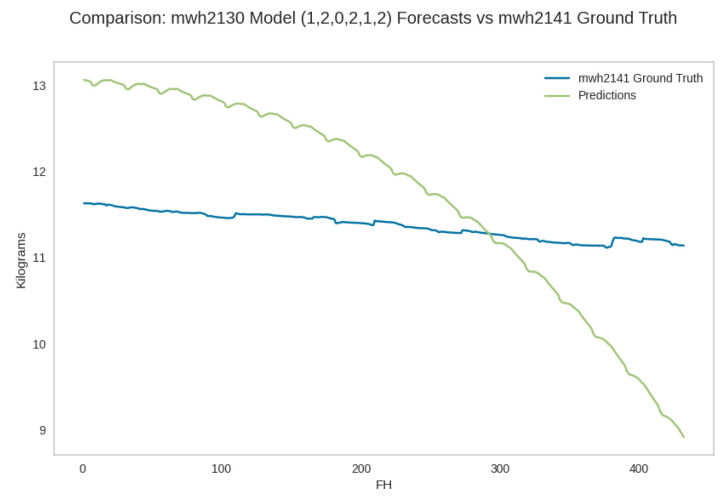
**Pattern 5: forecast failure.** The performance of the top MAPE 2130 p=1, d=2, q=0, P=2, D=1, Q=2 forecaster on the 2141 test data. The forecast values fail to reflect the trend and deviate from the actual ground truth values. The abbreviation *mwh* in the plot stands for *mean weight of hive*. The PDF may have to be enlarged to see the plot legends.

Figure A5,Figure A6,Figure A7,Figure A8,Figure A9 show the five most common weight forecast patterns we discovered in analyzing the performance of the top forecasters over the forecast horizon of 432 h. We will discuss these patterns in the next section. The supplementary materials contain all generalizability plots for the top RMSE and MAPE weight forecasters and the classification of patterns specific to the top RMSE weight forecasters. The reason why we only retained the patterns of the top RMSE weight forecasters is that, as discussed below, there was little or no difference between RMSE and MAPE as weight forecaster selection metrics.

#### 4.2.2. Temperature Forecasts

Table 5 gives the top three native RMSE and MAPE temperature forecasters for each hive found in the grid search. Figure A18 gives generalizability plots of the top RMSE 2059 weight forecaster on the test data of all ten hives. Figure A19 gives generalizability plots of the top MAPE 2059 weight forecaster on test data of all ten hives. The supplementary materials give generalizability plots of the top in-hive temperature forecasters of all ten hives.

**Figure A10:**
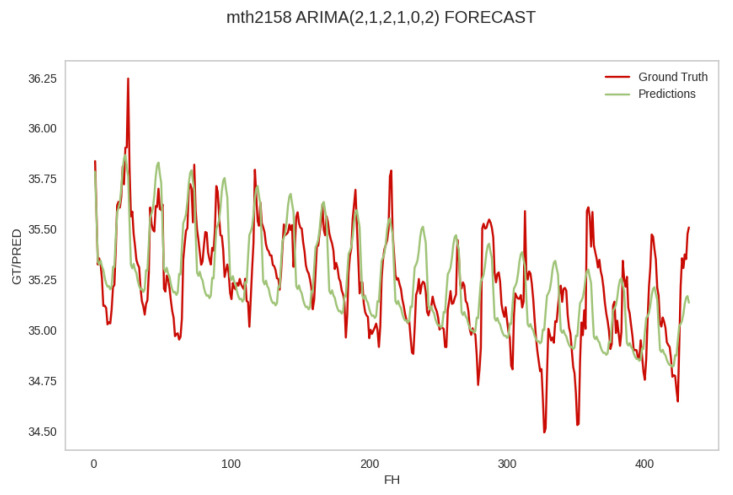
**Pattern 1: coincidence.** The performance of the third best RMSE 2158 forecaster p=2, d=1, q=2, P=1, D=0, Q=2 on the test data from the same hive. The forecast values coincide, more or less closely, with the ground truth vales and reflect the ground truth trend. The abbreviation *mth* in the plot stands for *mean temperature of hive*. The PDF may have to be enlarged to see the plot legends.

**Figure A11:**
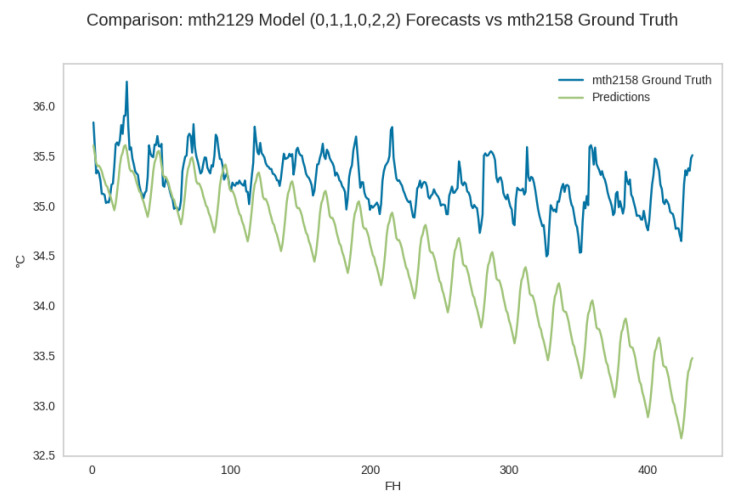
**Pattern 2: partial coincidence.** The performance of the top MAPE 2129 forecaster p=0, d=1, q=1, P=0, D=2, Q=2 on the test data for hive 2158. The forecast values coincide, more or less closely, with the ground truth vales and reflect the ground truth trend only for the initial segment of the forecast horizon but fail to reflect the trend and underestimate the ground truth observations for the remainder of the horizon. The abbreviation *mth* in the plot stands for *mean temperature of hive*. The PDF may have to be enlarged to see the plot legends.

**Figure A12:**
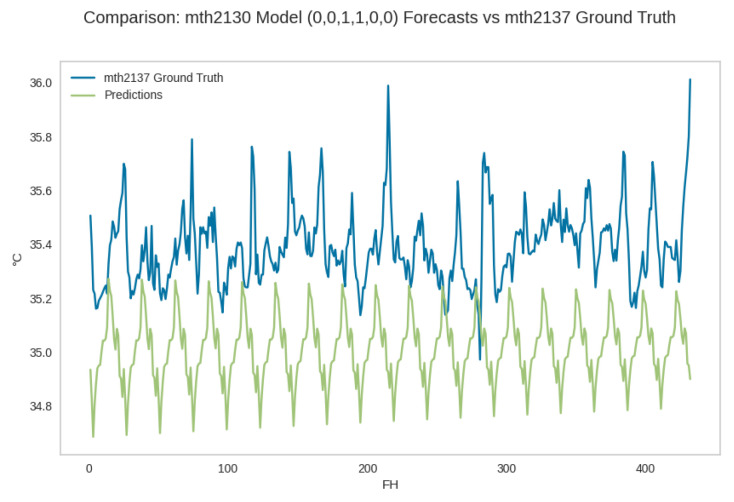
**Pattern 3: trend coincidence.** The performance of the second best MAPE forecaster p=0, d=0, q=1, P=1, D=0, Q=0 for hive 2130 on the test data of hive 2137. The forecast values reflect the ground truth trend, but underestimate the ground truth observations. The abbreviation *mth* in the plot stands for *mean temperature of hive*. The PDF may have to be enlarged to see the plot legends.

**Figure A13:**
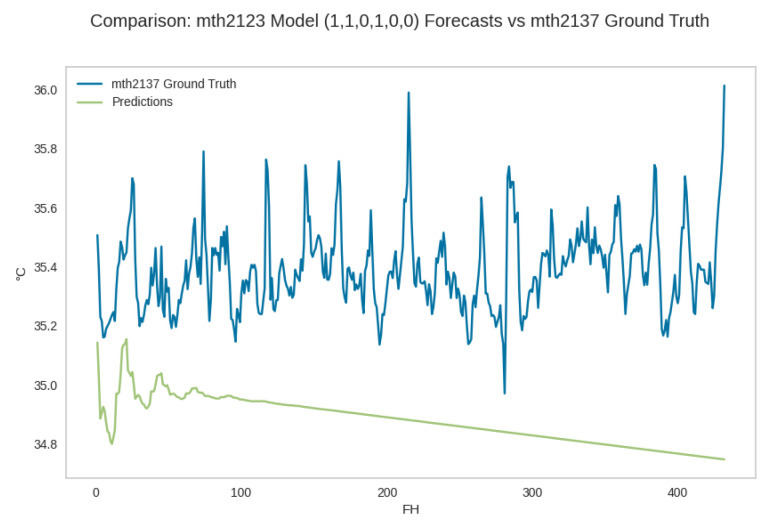
**Pattern 4: partial trend failure and coincidence.** The performance of the second best MAPE forecaster p=1, d=1, q=0, P=1, D=0, Q=0 for hive 2123 on the test data for hive 2137. The forecast values reflect the trend for the initial segment of the forecast horizon but fail to reflect the trend for the remainder of the horizon; the forecast also underestimates the ground truth observations for the entire horizon. The abbreviation *mth* in the plot stands for *mean temperature of hive*. The PDF may have to be enlarged to see the plot legends.

**Figure A14:**
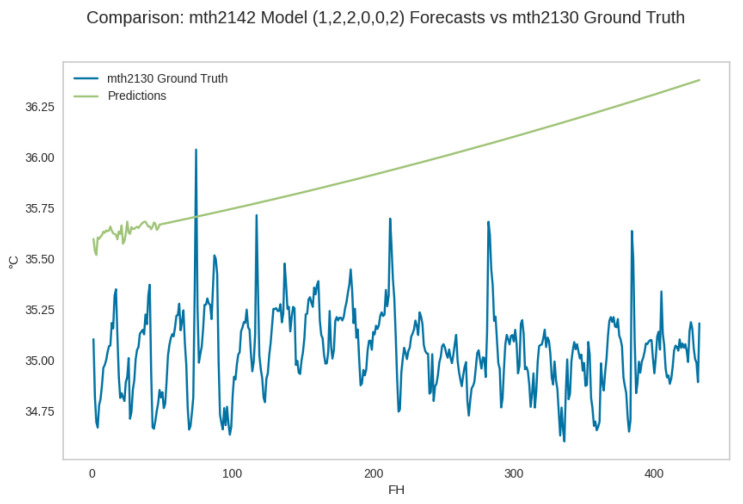
**Pattern 5: forecast failure.** The performance of the second best MAPE 2142 forecaster p=1, d=2, q=2, P=0, D=0, Q=2 on the 2130 test data. The forecast values fail to reflect the trend and overestimate the actual ground truth observations. The abbreviation *mth* in the plot stands for *mean temperature of hive*. The PDF may have to be enlarged to see the plot legends.

**Figure A15:**
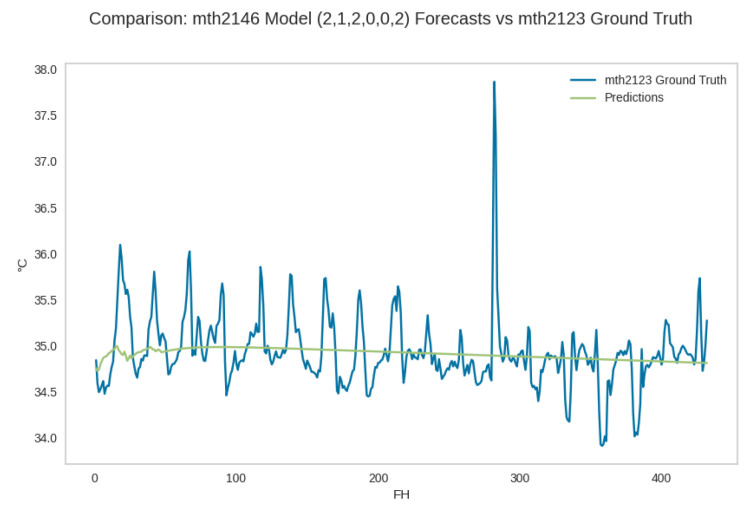
**Pattern 6: moving average trend.** The performance of the top RMSE and MAPE forecaster p=2, d=1, q=2, P=0, D=0, Q=2 for hive 2146 on the test data for hive 2123. The forecast values accurately reflect the ground truth trend, while approximating to the mean of the series. The abbreviation *mth* in the plot stands for *mean temperature of hive*. The PDF may have to be enlarged to see the plot legends.

**Figure A16:**
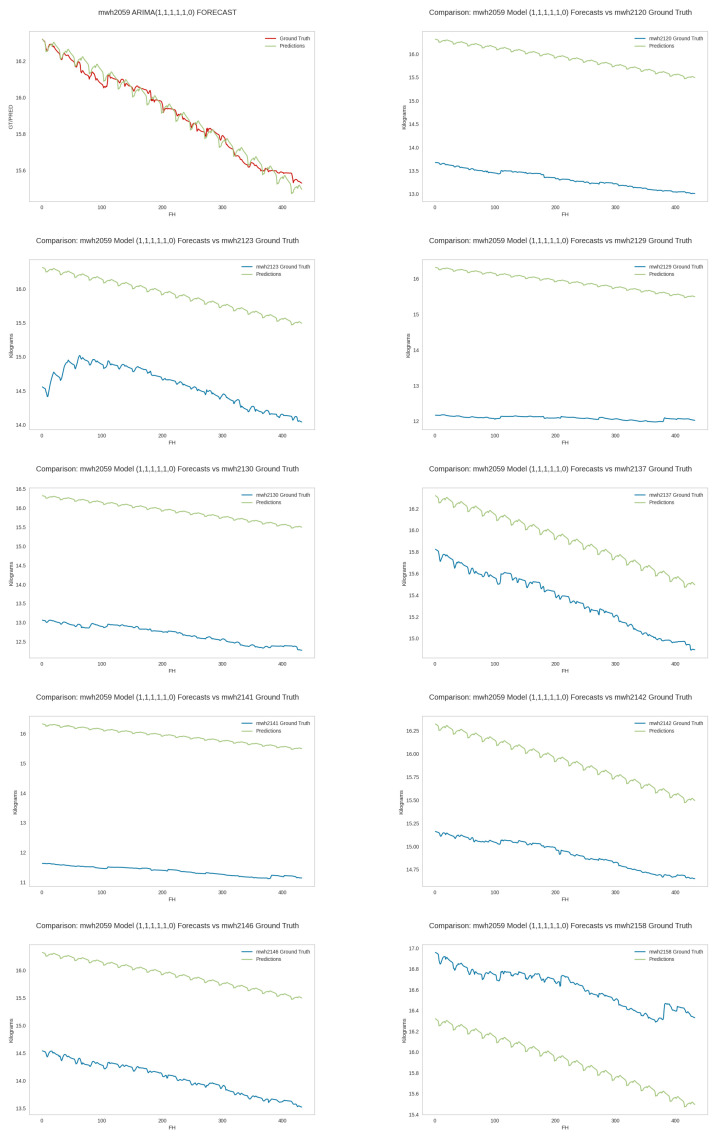
**Generalizability of top RMSE 2059 weight forecaster.** The performance of the top RMSE model for hive 2059 on the test data of all ten hives. The PDF should be enlarged to see the details.

**Figure A17:**
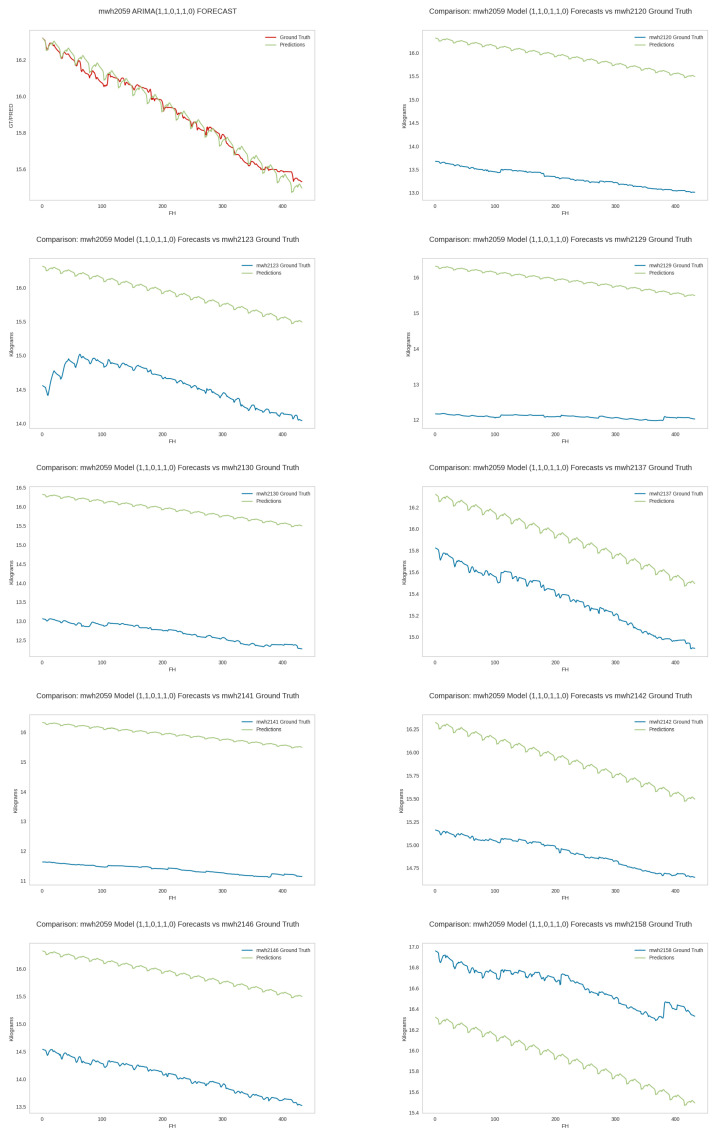
**Generalizability of top MAPE 2059 weight forecaster.** The performance of the top MAPE forecaster for hive 2059 on the test data of all ten hives. The PDF should be enlarged to see the details.

**Figure A18:**
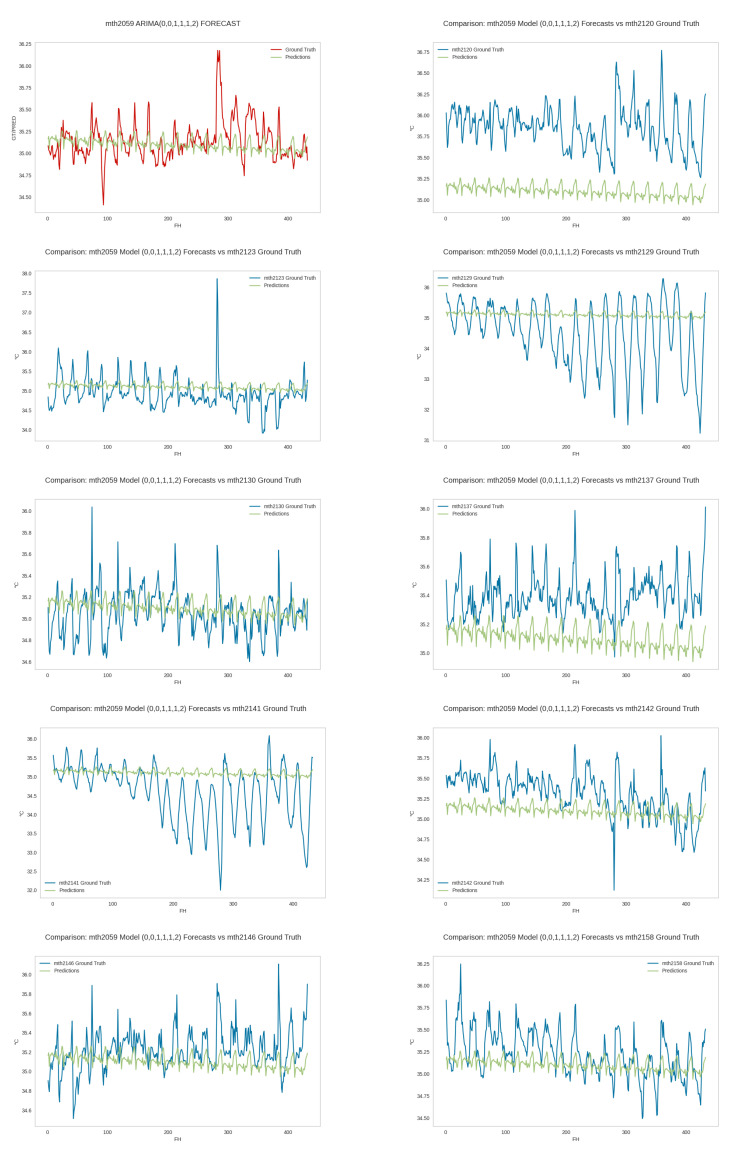
**Generalizability of top RMSE 2059 in-hive temperature forecaster.** The performance of the top RMSE temperature forecaster for hive 2059 on the test data of all ten hives. The PDF should be enlarged to see the details.

**Figure A19:**
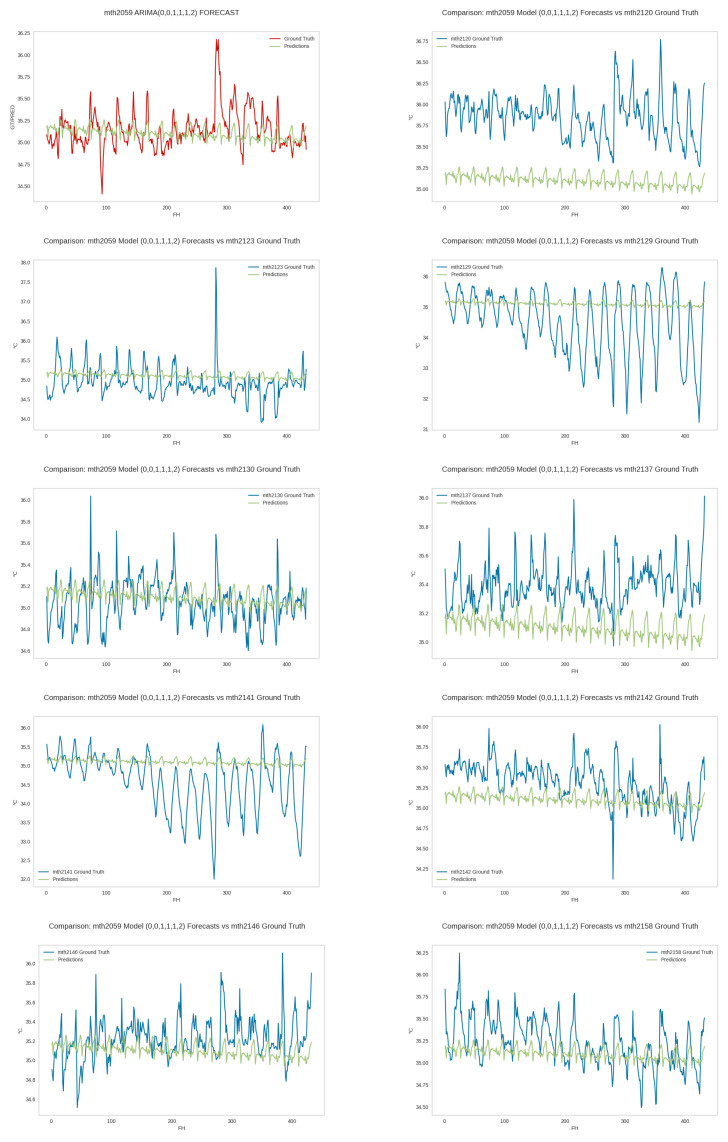
**Generalizability of top MAPE 2059 in-hive temperature forecaster.** The performance of the top MAPE temperature forecaster for hive 2059 on the test data of all ten hives. The PDF should be enlarged to see the details.

Figure A10,Figure A11,Figure A12,Figure A13,Figure A14,Figure A15 demonstrate the six most common in-hive temperature forecast patterns we observed in analyzing the performance of the top forecasters over the 432 h of the temperature observations. We will discuss these and other minor patterns qualitatively and quantitatively in the next section. The supplementary materials contain all generalizability plots for the top RMSE and MAPE temperature forecasters and the classification of patterns specific to the top RMSE temperature forecasters. The reason why we only retained the patterns of the top RMSE temperature forecasters was the same as for the RMSE weight forecasters: there was little or no difference between RMSE and MAPE as temperature forecaster selection metrics.

### 4.3. Weight and Temperature Patterns

In analyzing the performance of the top forecasters, we discovered six forecast patterns, of which five appeared to apply to weight and temperature, and one appeared to be temperature-specific. We gave the following descriptive names to the five shared patterns: (1) *coincidence*; (2) *partial coincidence*; (3) *trend coincidence*; (4) *partial trend failure and coincidence*; (5) *forecast failure*. We called the sixth temperature-specific pattern *moving average trend*.

The coincidence pattern (Pattern 1) (cf. Figure A5 and Figure A10) occurs when a forecaster accurately predicts the actual ground truth observations and their trend (upward, downward, or flat) over the entire forecast horizon for a hive. This is the best forecast scenario. A partial coincidence (Pattern 2) (cf. Figure A6 and Figure A11) occurs when a forecaster accurately predicts the ground truth observations and the trend over a significant segment of a forecast horizon and deviates from the ground truth observations (but not the trend!) over the remainder of the horizon. This is the second best forecast scenario. We arbitrarily chose the length of the segment to be at least one hundred hours (i.e., the forecaster’s predictions must coincide with the ground truth observations and trend for at least one hundred observations over a given forecast horizon). Trend coincidence (Pattern 3) (cf. Figure A7 and Figure A12) occurs when a forecaster accurately reflects the trend in a hive over the entire forecast horizon but either underestimates or overestimates it. Depending on the objectives of a specific forecasting application, this could be the best, second best, or third best scenario. For example, if a stakeholder’s objective is to only predict trends over entire forecasting horizons, it is the best scenario. Partial trend failure and coincidence (Pattern 4) (cf. Figure A8 and Figure A13) is exhibited by a forecaster that fails to predict a trend for a hive over a segment of the forecast horizon and exhibits Pattern 3 over the remainder of the horizon. This is the fourth best scenario, in that the forecaster can at least predict the trend for a segment of the forecasting horizon. The forecast failure pattern (Pattern 5) (cf. Figure A9 and Figure A14) is exhibited by a forecaster that fails to predict the ground truth observations and the trend. This is the worst scenario: the forecaster is useless.

A temperature-specific moving average trend (Pattern 6) (cf. Figure A15) occurs when a forecaster accurately captures the general trend (upward, downward, or flat) of the temperature series around its central tendency over the entire forecast horizon. However, the forecast systematically fails to predict the actual ground truth observations, in that it fails to reflect short-term cyclical variations, such as peaks and troughs. Thus, the moving average trend forecast has a smoothed trajectory that aligns with the long-term trend, but deviates from the finer-scale fluctuations observed in the ground truth data. We called this pattern *moving average trend*, because it resembles a simple moving average effect, where the forecast tracks the overarching trend but lacks the precision to predict periodic deviations from it.

Table 6 and Table 7 document the pattern frequencies of the native and non-native forecasts of the best RMSE weight and temperature forecasters.

## 5. Discussion

Our study had two main goals: (1) Explore the stationarity, trend, seasonality, and distribution of the hive weight and in-hive temperature of ten managed honey bee colonies at a research apiary, where the hives were monitored with electronic scales and in-hive temperature sensors from June to October 2022; (2) If the exploration showed that the series were not white noise and not normally distributed, perform a systematic autoregressive integrated moving average (ARIMA) time series analysis to answer three fundamental questions: (a) Does seasonality matter in the ARIMA forecasting of hive weight and in-hive temperature? (b) To what extent do the best forecasters of one hive generalize to other hives? and (c) Which time series type (i.e., hive weight or in-hive temperature) is better predictable?

The exploratory analysis indicated that the hive weight and in-hive temperature series were not white noise and not normally distributed. Both types of time series for most hives were not difference- or trend-stationary. Thus, the ARIMA and SARIMA time series forecasting exploitation of the datasets was justified.

The grid search of 769 weight and 769 temperature forecasters (cf. Table 4 and Table 5) showed that seasonality mattered over a forecast horizon of 432 h. As a comparison baseline, non-seasonal ARIMA was outperformed by SARIMA over this horizon. In particular, Table 4 shows that only 2 out of the top 60 weight forecasters (i.e., 3.3%) found in the grid search had the three seasonality parameters *P*, *D*, *Q* equal to 0. Of the top 60 temperature forecasters, only one forecaster (i.e., 1.7%) had zero seasonality parameters. This result complements the findings in [27], which was Part I of our investigation, where non-seasonal ARIMA performed on par with artificial neural networks, convolutional neural networks, and long short-term memory models in predicting trends for shorter horizons from 6 up to 24 h. In Part I of our investigation, non-seasonal ARIMA was used as the comparison baseline and was not outperformed by the machine learning methods for shorter horizons.

The distribution of patterns in the native and non-native weight and temperature forecasts (cf. Table 6 and Table 7) indicate that, on the curated dataset, the forecasters were better at predicting the weight and temperature of the native hives. Among the native weight forecasts, four of the best forecasters predicted the ground truth values and trends completely and one forecaster predicted them partially; five forecasters successfully predicted trends. There were no trend or forecast failures. The situation was reversed for the non-native weight forecasts, where no forecasts predicted observations and trends completely or partially.

We observed a similar picture in the native temperature forecasts. Among the native forecasts, five native forecasts predicted the ground truth observations and trends either completely or partially, while four native forecasts successfully predicted the trends. There was also one instance of a moving average trend, and no forecast failures were observed. Among the non-native forecasts, the majority—69 forecasts (77.8%)—accurately predicted trends; if we consider the moving average trend patterns as also predicting trends, the number of non-native forecasts predicting trends rises to 79 (87.8%).

Table 4 and Table 5 show that the RMSE and MAPE scores of the top three weight forecasters were lower than those of the top three for temperature. This result indicates that weight may be more accurately predictable than temperature, which, in turn, may be explained by the fact that weight fluctuated less than temperature across all monitored hives.

The forecasters selected by RMSE and MAPE (cf. Figure A3 and Figure A4) showed very similar predictions over the entire forecast horizon. Thus, either metric can be used to select the best forecaster on the curated dataset. The results of the top three native hive weight forecasters in Table 4 indicate that practical applications of SARIMA to forecast native hive weight may use a threshold between 0.02 and 0.08 for RMSE and between 0.0015 and 0.015 for MAPE over a horizon of 432 h. The results of the top three native in-hive temperature forecasters in Table 5 indicate that practical applications of SARIMA to forecast native in-hive temperature may use a threshold between 0.12 and 0.89 for RMSE and a threshold between 0.002 and 0.02 for MAPE over the horizon of 432 h. Of course, these recommendations should be taken with caution, because ambient weather, exposure to pesticides, and queen lines may influence the colony’s weight and thermoregulation patterns. Further longitudinal research is required to find an optimal placement of in-hive temperature sensors in a Langstroth hive. As the reviewed hive monitoring literature suggests, in-hive temperature can be affected by ambient temperature, depending on where the temperature sensor is placed. Bees do not control temperature very much in the outer frames, so there may be an interaction between ambient temperature and bee-controlled temperature. However, if one were to monitor temperature at the center of the brood cluster, the literature indicates that very little impact of ambient temperature is found (see, e.g., [15]).

Our approach has several limitations. The weight monitoring of the hives was confined to the hives with exactly two Langstroth supers. Consequently, hives with more than two supers may exhibit different weight patterns. The temperature monitoring was carried out with temperature sensors placed at the top bar of the middle frame in the second deep super of each hive. The in-hive temperature literature indicates that in-hive temperature may be affected by the location of the temperature sensor in the hive (see, e.g., [15,16]). Thus, a study where in-hive temperature sensors are placed in a different location may exhibit different (e.g., more or less predictable) time series patterns. Some investigations show that colony performance and health depends on ambient weather (see, e.g., [1,25]) and on geographical locations, e.g., the foraging distance proximity of apiaries to the U.S. Conservation Reserve Program lands [14]. Thus, the predictability of hive weight and in-hive temperature may be affected by the geographical location of monitored apiaries.

While ambient weather, geographical location, pesticide exposure, and other factors influence a colony’s hive weight and in-hive temperature, they may not necessarily influence the predictability of the weight or temperature observations obtained from deployed sensors. A weight forecaster built on weight observations from a hive in an arid, dry climate where moisture does not impact the weight of the woodenware will necessarily be different from a weight forecaster built on the observations from a hive in a humid, rainy climate. Analogously, a temperature forecaster fitted on the observations from a hive in a warm climate will be different from a temperature forecaster trained on the observations from a hive in a colder climate. That said, all forecasters should display some degree of predictability, so long as the training data are not white noise. Therefore, precision pollination researchers and citizen scientists can use the presented methods to analyze hive weight and in-hive temperature observations. In particular, if exploration of the observations from a particular hive indicates that they are not random, our grid search method can be used to identify hive weight and in-hive temperature forecasters for monitored hives.

We expect our methods to work with any off-the-shelf electronic scale or in-hive temperature sensor capable of logging digital measures at user-specified intervals (e.g., every 5 min). The data can then be easily converted into the CSV format, similar to the format in our datasets in the supplementary materials. Once this is achieved, our grid search code can be used to find the best forecasters. Designers of electronic scales and in-hive temperature sensors can benefit from our approach,—provided their data loggers connect to a cloud computing and storage service—since real-time exploration and grid-search exploitation of the captured time-series data enable the discovery of optimal forecasters.

## 6. Conclusions

Our study indicates that hive weight and in-hive temperature time series are not white noise and are not normally distributed. Most of them are not difference- or trend-stationary. Thus, it is possible to exploit time series data to identify optimal ARIMA/SARIMA hive weight and in-hive temperature with parameterized grid searches. For the investigated forecast horizons of 432 h, seasonality mattered, in that most of the top three forecasters had at least one non-zero seasonality parameter. Native forecasts (i.e., the forecasts made by the forecasters for the hives on which they were trained) were more accurate than non-native forecasts, which indicates that forecasters may not generalize well to predict the weight or temperature of other hives. An alternative way to interpret this finding is to hypothesize that colonies differ in their weight and temperature characteristics, even when they have the same queen lines and are hived in the same apiary. However, this hypothesis requires more longitudinal investigations and a much broader sharing of digital hive inspections executed according to commonly accepted protocols. When applied to non-native hives, most hive weight and in-hive temperature forecasters accurately predicted trends. Of the two hive characteristics investigated in our study (i.e., hive weight and in-hive temperature), hive weight was more accurately predicted than in-hive temperature by the top three forecasters found in the grid search. We hope that precision apiculture researchers and practitioners can replicate our results and use our ARIMA/SARIMA grid search to create custom hive weight or in-hive temperature forecasters for their investigations of colony phenology.

## Figures and Tables

**Table 1:** **Hive weight and in-hive temperature means for hour 10 on 7 July 2022.** The means for hour 10 were computed from the 12 hive weight and in-hive temperature observations from 9:00 up to 9:55. Table legend: HID—USDA-ARS Hive ID; HR—hour (a non-negative integer used instead of a time stamp); μW—mean hive weight (kg) for hour 10; μC—mean in-hive temperature (degrees Celsius) for hour 10.

HID	HR	μW	μC
2059	10	15.70	35.38
2120	10	15.76	35.93
2129	10	14.79	35.89
2123	10	14.84	35.16
2130	10	14.04	35.36
2137	10	16.41	35.38
2141	10	13.55	35.12
2142	10	13.74	35.22
2146	10	16.01	35.48
2158	10	16.50	35.58

**Table 2:** **Exploratory hive weight summary.** This table gives the results obtained with the Ljung–Box White Noise, Augmented Dickey–Fuller, Kwiatkowski–Phillips–Schmidt–Shin, and Shapiro–Wilk Normality tests we used in our exploratory analysis of the hive weight data.

Statistic	Value
Total number of observations	2160 for all hives
Seasonality Type	multiplicative for all hives
Primary seasonality	24 for all hives, except for 2141 (13)
Recommended d	d = 1 for all hives, except for 2142 (d = 2)
Recommended D	0 for all hives
Ljung-Box White Noise	false for all hives
Shapiro-Wilk Normality	false for all hives
ADF	false for all hives, except for 2142
KPSS	false for all hives

**Table 3:** **Exploratory in-hive temperature summary.** The table gives the results obtained with the Ljung–Box White Noise, Augmented Dickey–Fuller, Kwiatkowski–Phillips–Schmidt–Shin, and Shapiro–Wilk Normality tests we used in our exploratory analysis of the in-hive temperature data.

Statistic	Value
Total number of observations	2160 for all hives
Seasonality Type	Multiplicative for all hives
Primary seasonality	24 for all hives, except for 2137 (48)
Recommended d	d = 1 for all hives
Recommended D	0 for all hives
Ljung–Box White Noise	false for all hives
Shapiro–Wilk Normality	false for all hives
ADF	2120, 2123, 2137, 2142, 2146—true;
	2059, 2129, 2130, 2141, 2158—false
KPSS	false for all hives, except for 2130 (true)

**Table 4:** **Top three native RMSE and MAPE weight forecasters found in the grid search.** The three forecasters with the zero seasonality parameters, of which two are identical, are bolded. RMSE and MAPE values are to the left of the corresponding forecasters.

HID	Top N	RMSE	p	d	q	P	D	Q	MAPE	p	d	q	P	D	Q
2059	1	0.0338	1	1	1	1	1	0	0.0017	1	1	0	1	1	0
	2	0.0338	0	1	2	1	1	0	0.0017	2	1	0	1	1	0
	3	0.0339	1	0	1	1	0	2	0.0017	1	1	1	1	1	0
2120	1	0.0258	2	1	0	1	0	2	0.0015	**1**	**2**	**0**	**0**	**0**	**0**
	2	0.0259	0	1	2	2	0	2	0.0016	2	1	0	1	0	2
	3	0.0262	0	1	1	1	0	2	0.0016	2	1	1	2	0	0
2123	1	0.2160	1	2	0	2	1	2	0.0126	1	2	0	2	1	2
	2	0.2511	**2**	**2**	**1**	**0**	**0**	**0**	0.0137	**2**	**2**	**1**	**0**	**0**	**0**
	3	0.2557	0	0	2	2	0	1	0.0153	0	0	2	2	0	1
2129	1	0.0393	1	1	2	2	2	1	0.0028	1	1	2	2	2	1
	2	0.0456	1	1	1	2	2	1	0.0031	1	1	1	2	2	1
	3	0.0529	0	1	0	2	2	2	0.0035	0	1	0	2	2	2
2130	1	0.0544	1	2	0	2	1	2	0.0033	1	2	0	2	1	2
	2	0.0624	1	2	0	2	1	0	0.0037	1	2	0	2	1	0
	3	0.0649	2	0	2	1	2	2	0.0042	2	0	2	2	2	1
2137	1	0.0420	0	1	1	1	1	0	0.0022	2	1	0	1	1	0
	2	0.0424	2	1	1	1	1	0	0.0022	0	1	1	1	1	0
	3	0.0427	2	1	0	1	1	0	0.0022	2	1	1	1	1	0
2141	1	0.0336	1	1	0	2	0	2	0.0024	1	1	2	2	0	2
	2	0.0336	0	1	0	2	0	2	0.0024	2	1	2	1	0	2
	3	0.0336	0	1	1	2	0	2	0.0024	1	1	2	0	0	2
2142	1	0.0272	1	2	2	2	0	0	0.0015	1	2	2	2	0	0
	2	0.0346	0	1	2	0	2	1	0.0018	0	1	2	0	2	1
	3	0.0402	1	0	0	1	0	2	0.0022	0	1	0	0	2	2
2146	1	0.0403	0	1	1	2	0	1	0.0023	0	1	1	2	0	1
	2	0.0413	0	2	2	0	0	2	0.0023	0	2	2	0	0	2
	3	0.0428	2	1	1	1	1	1	0.0024	1	1	0	2	1	1
2158	1	0.0789	2	1	2	0	1	0	0.0038	2	1	1	0	1	0
	2	0.0809	1	1	2	0	1	0	0.0039	1	1	2	0	1	0
	3	0.0816	2	1	0	0	1	0	0.0039	2	1	0	0	1	0

**Table 5:** **Top three native RMSE and MAPE temperature forecasters found in the grid search.** RMSE and MAPE values are to the left of the corresponding forecasters. The only forecaster with zero seasonality coefficients is bolded.

HID	Top N	RMSE	p	d	q	P	D	Q	MAPE	p	d	q	P	D	Q
2059	1	0.2200	2	0	2	1	0	2	0.0045	0	0	1	1	1	2
	2	0.2235	1	0	2	2	0	0	0.0045	2	1	1	1	0	0
	3	0.2237	0	0	1	1	1	2	0.0046	0	0	1	2	1	1
2120	1	0.2318	1	1	2	1	0	1	0.0047	1	1	2	1	0	1
	2	0.2320	1	1	1	2	0	1	0.0048	1	1	1	2	0	1
	3	0.2342	1	1	2	2	0	1	0.0048	2	1	1	1	0	1
2123	1	0.3628	0	0	2	1	0	0	0.0069	0	0	2	1	0	0
	2	0.3709	1	1	2	1	1	2	0.0069	1	1	0	1	0	0
	3	0.3723	1	1	0	1	0	0	0.0069	2	1	0	1	0	0
2129	1	0.8370	0	1	1	0	2	2	0.0191	0	1	1	0	2	2
	2	0.8638	1	1	1	0	2	2	0.0197	1	1	1	0	2	2
	3	0.8900	0	1	0	0	2	2	0.0202	0	1	0	1	2	2
2130	1	0.1815	0	0	1	2	1	1	0.0038	0	0	1	2	1	1
	2	0.1826	0	0	2	2	1	1	0.0038	0	0	1	1	0	0
	3	0.1827	0	0	1	2	1	2	0.0039	2	0	0	2	1	2
2137	1	0.1208	2	1	0	1	0	1	0.0026	2	1	0	1	0	1
	2	0.1225	2	1	0	0	1	1	0.0027	0	1	0	1	1	2
	3	0.1225	0	1	2	0	1	1	0.0027	0	1	0	1	1	1
2141	1	0.5968	2	1	2	0	2	2	0.0129	2	1	2	0	2	2
	2	0.6122	1	0	0	2	2	0	0.0131	1	1	2	0	2	2
	3	0.6128	1	1	2	0	2	2	0.0132	1	0	0	2	2	0
2142	1	0.1945	2	1	1	1	1	2	0.0040	2	1	1	1	1	2
	2	0.2028	2	1	1	0	1	1	0.0043	1	2	2	0	0	2
	3	0.2072	**2**	**2**	**2**	**0**	**0**	**0**	0.0043	2	2	1	1	0	2
2146	1	0.2027	2	1	2	0	0	2	0.0047	2	1	2	0	0	2
	2	0.2197	0	0	1	1	0	0	0.0049	0	0	1	1	0	0
	3	0.2245	2	0	0	1	0	2	0.0051	2	0	0	1	0	2
2158	1	0.1953	2	1	0	2	0	1	0.0045	2	1	0	2	0	1
	2	0.2041	2	1	1	2	0	1	0.0045	2	1	1	2	0	1
	3	0.2048	2	1	2	1	0	2	0.0046	0	1	2	2	0	1

**Table 6:** **Frequency of qualitative classification patterns of the best RMSE weight forecasters in the native and non-native forecasts.** A forecaster’s native hive is the hive on whose training data the forecaster was fitted. LEGEND: NF—Native Frequency (pattern frequency in native weight forecasts); NNF—Non-Native Frequency (pattern frequency in non-native weight forecasts).

	Pattern	NF	NNF
1	Coincidence	4	0
2	Partial Coincidence	1	0
3	Trend Coincidence	5	80
4	Partial Trend Failure and Coincidence	0	8
5	Forecast Failure	0	2
	Total	10	90

**Table 7:** **Frequency of qualitative classification patterns of the best RMSE temperature forecasters in the native and non-native forecasts.** A forecaster’s native hive is the hive on whose training data the forecaster was fitted. LEGEND: NF—Native Frequency (pattern frequency in native temperature forecasts); NNF—Non-Native Frequency (pattern frequency in non-native temperature forecasts).

	Pattern	NF	NNF
1	Coincidence	3	0
2	Partial Coincidence	2	5
3	Trend Coincidence	4	69
4	Partial Trend Failure and Coincidence	0	0
5	Forecast Failure	0	6
6	Moving Average Trend	1	10
	Total	10	90

**Figure A2:**
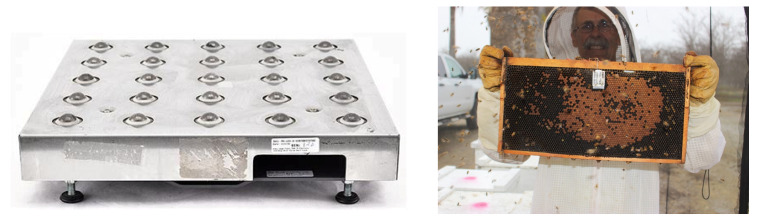
**Weight and temperature sensors.** (**Left**): Tekfa model B-2418 electronic scale (precision: ±20 g; operating temperature: −30°C to 70 °C) linked to a 16-bit datalogger (Hobo UX120-006M External Channel data logger, Onset Computer Corporation, Bourne, MA, USA). (**Right**): An internal wireless temperature sensor (Hobo MX2201 sensor, Onset Computer Corporation, Bourne, MA, USA; accuracy ± 0.5 °C) placed at the top bar of the middle frame in the second (higher) box of each monitored hive. The scales were powered from the grid. Each in-hive temperature sensor had its own battery the size of a small coin that can power it for ≈12 months.

## Data Availability

The supplementary materials are available at [23]. The materials include our source code, curated datasets, performance plots, and performance CSV logs.

## Abbreviations

ARIMA	Autoregressive Integrated Moving Average
SARIMA	Seasonal ARIMA
ADF	Augmented Dickey–Fuller
KPSS	Kwiatkowski–Phillips–Schmidt–Shin
RMSE	Root Mean Squared Error
MAPE	Mean Absolute Percentage Error
CSV	Comma Separated Values
KNN	K Nearest Neighbors
USDA	U.S. Department of Agriculture
ARS	Agricultural Research Service
